# Cross-cultural differences in visual object and background processing in the infant brain

**DOI:** 10.1162/imag_a_00038

**Published:** 2023-12-13

**Authors:** Moritz Köster, Anna Bánki, Daiki Yamasaki, Masaharu Kato, Shoji Itakura, Stefanie Hoehl

**Affiliations:** Institute of Psychology, University of Regensburg, Regensburg, Germany; Department of Psychology, Kyoto University, Kyoto, Japan; Faculty of Education and Psychology, Freie Universität Berlin, Berlin, Germany; Faculty of Psychology, University of Vienna, Vienna, Austria; Center for Baby Science, Doshisha University, Kizugawa, Japan

**Keywords:** visual system development, infant cognition, frequency tagging, cross-cultural comparison, social learning

## Abstract

Human visual cognition differs profoundly between cultures. A key finding is that visual processing is tuned toward focal elements of a visual scene in Western cultures (US and Europe) and toward the background in Eastern cultures (Asia). Although some evidence for cultural differences exists for young children, to date, the ontogenetic origins of cultural differences in human visual cognition have not been unveiled. This study explores early cross-cultural differences in human visual processing, by tracking the neural signatures for object versus background elements of a visual scene in the electroencephalogram (EEG) of 12-month-old infants, in Vienna (Austria; a Western culture; *n* = 35) and Kyoto (Japan; an Eastern culture; *n* = 36). Specifically, we separated neural signatures by presenting object and background at different stimulation frequencies (5.67 and 8.5 Hz). Results show that human visual processing is different between cultures from early on. We found that infants from Vienna showed a higher object signal, in contrast to infants from Kyoto, who showed an accentuated background signal. This early emergence of cultural differences in human vision may be explained in part by early social experiences: In a separate interaction phase, mothers from Vienna pointed out object (versus background) elements more often than mothers from Kyoto. To conclude, with a cross-cultural developmental neuroscience approach, we reveal that cross-cultural differences in visual processing of object and background are already present in the first year after birth, which is much earlier than previously thought.

## 1 Introduction

Human visual cognition differs profoundly between cultures (Henrich et al., 2010; Markus & Kitayama, 1991; Nisbett et al., 2001). For example, it has been established that individuals from Western cultures (e.g., the United States and Europe) show a perceptual bias for the objects in a visual scene, compared to individuals from Eastern cultures (e.g., China and Japan) who take into account contextual elements such as the background (and relations between elements) to a higher extent than Westerners (Nisbett & Masuda, 2003; Nisbett & Miyamoto, 2005)—a phenomenon coined context-sensitivity. These cultural differences in visual processing have been shown, for example, for verbal scene descriptions and memory encoding (Masuda & Nisbett, 2001), as well as gaze behavior (Chua et al., 2005) in adults.

Different explanatory accounts for cultural differences in object and background perception exist, grounding these differences in Western and Eastern cultural histories (Nisbett & Norenzayan, 2002), in affordances of the visual environment (Miyamoto et al., 2006), or the social orientation toward individualism versus collectivism in these cultures (Varnum et al., 2010). Recently, social interactions with others have been identified as a potential mechanism that shapes visual processing in early childhood (Jurkat et al., 2021, 2023; Köster & Kärtner, 2018; Senzaki & Shimizu, 2022; Senzaki et al., 2016), as well as human brain development more generally (Huber et al., 2023). These findings resonate with the crucial role of social interactions in early object learning, namely that children integrate social and object knowledge to allocate their attention to the environment from the infant years on (Pauen et al., 2015). In prior studies, the way parents guided their children’s attention to objects and background of visual scenes affected children’s visual perception from around the preschool years, which is also the age that cultural differences in context-sensitivity have first been reported using behavioral measures (Bremner et al., 2016; Imada et al., 2013).

However, the ontogenetic origins of when and how culture begins to shape human visual processing of object and background in a visual scene are not yet understood. A recent study suggests that cultural differences in parental guidance of visual attention may be present much earlier, when infants are 6 to 15 months old (Senzaki & Shimizu, 2020). Furthermore, from developmental neuroscience, we know that the visual system is shaped by perceptual experience from birth (Berardi et al., 2000). For example, this has been shown in the mammalian brain, by testing the effect of environmental deprivation (Blakemore & Cooper, 1970), as well as in human infants, by studying the effects of abnormal binocular experience (Banks et al., 1975) or early top-down effects on the visual system (Emberson et al., 2015). This raises the crucial question, at which age lived experiences that infants make in their specific socio-cultural environment begin to impact the development of their visual system.

Several lines of research speak for an early influence of the cultural context on visual processing in social interaction with others (Astor et al., 2022; Bard et al., 2021; Haensel et al., 2020; Kärtner et al., 2010; Keller, 2007) and for social stimuli (Geangu et al., 2016; Green et al., 2016). For example, by 2 months of age, the dyadic gaze patterns between mothers and their infants show different developmental trajectories between cultures (Kärtner et al., 2010), which may lay the ground for cultural differences in joint attention engagement toward the end of the first year (Astor et al., 2022; Bard et al., 2021). Concerning social stimuli, cultural differences have been described between infants from a Central European and an East Asian background, with regards to the visual scanning of faces (Geangu et al., 2016) and action prediction (Green et al., 2016). In a study with 8-month-olds, Chinese infants only predicted the goal of eating actions performed with chopsticks, whereas Swedish infants exclusively predicted the goal of eating actions performed with a spoon (Green et al., 2016). However, to the best of our knowledge, early cross-cultural differences in the visual processing of the physical world, such as the processing of visual scenes with object and background, have not been investigated in infancy, thus far.

One limitation of former research on visual object and background processing has been the focus on verbal and behavioral outcome measures of context-sensitivity Köster et al., 2018; Mavridis et al., 2020; Senzaki et al., 2016. Thus, our understanding of the development of cross-cultural differences may be obscured by young children’s linguistic proficiency or memory capacities. This limitation can be overcome by assessing the visual processing of object and background more directly, at a neurophysiological level. A highly useful method from cognitive neuroscience that allows assessing context-sensitive processing in the visual system is frequency tagging (Köster et al., 2023; Müller et al., 2003; Norcia et al., 2015; Vialatte et al., 2010): presenting object and background of a visual scene periodically (switching the presentation on and off rapidly) at different driving frequencies elicits distinct signatures for elements of a visual scene (e.g., object and background) in visual cortical networks, which can be assessed in the EEG (Köster et al., 2017; Martens et al., 2011). Frequency tagging is a particularly promising tool to explore context-sensitive processing in the infant brain, given the possibility of tracking object and background simultaneously at the neural process level, its high signal-to-noise ratio, and the capacity to capture both overt and covert visual attention (Christodoulou et al., 2018; Robertson et al., 2012).

In the present study, based on early cross-cultural differences in visual system development in the social domain and initial evidence that culture-specific social guidance of object and background processing are present from very early in life, we hypothesized that the socio-cultural environment may shape human visual object and background processing, beginning in the first year, just after infants begin to follow the attention guidance by others (Tomasello et al., 2005). We applied frequency tagging to assess the processing of object and background of natural visual scenes in 12-month-olds from Vienna (Austria; *n* = 35) and Kyoto (Japan; *n* = 36). At this age, infants have just begun to reliably follow parental guidance of attention, pointing to objects in a picture book (which we established in a piloting phase of the present study). In an interaction phase, we assessed maternal spontaneous pointing to object or background, to identify cultural differences in early social experiences as a potential shaping mechanism of visual perception (Csibra & Gergely, 2009).

## 2 Methods

### 2.1 Participants

Participants were 71 full-term born infants with typical development from Vienna, Austria (*n* = 35; age in months: *M* = 11.98, *SD* = 0.5, *range* = 11.11-13.02; 20 girls) and Kyoto, Japan (*n* = 36; age in months: *M* = 12.13, *SD* = 0.74, *range *=10.95-13.45; 22 girls), and their mothers (Vienna: age in years: *M *=35.14, *SD *=4.57; Kyoto: age in years: *M *=33.84, *SD *=4.56). Families were recruited via participant databases at both study sites. Note that all Austrian (*n *=35) but not all Japanese (*n *=32) families provided demographic and questionnaire data (leading to a reduced sample reported in this section). Ages were similar for infants and mothers across cultures, respectively, *t*(65)=-0.94, *p *=.349, *d *=-0.23, and, *t*(65)=1.16, *p *=.249, *d *=0.29. The study was approved by the Ethics Committee of the University of Vienna (Ref. 00382) and Doshisha University (Ref. 19017). Informed written consent prior to participation in the study was obtained from the mother.

To establish whether the two groups were culturally different, we assessed parental socialization goals with the Keller scale (Keller, 2007). This scale captures caregivers’ attitudes toward autonomous or relatedness socialization goals reflecting autonomous and relational cultural prototypes. Caregivers needed to rate how important each socialization goal (e.g., autonomous goal: “developing own interests,” relatedness goal: “helping others”) is for their child to achieve by the age of three, on a 4-point Likert scale. We found a higher emphasis on autonomous socialization goals in Vienna compared to Kyoto (Vienna: *M *=3.6, *SD *=0.4; Kyoto: *M *=3.3, *SD *=0.4), *t*(65)=2.90, *p *=.005, *d *=0.70, but there was no significant difference in the emphasis on relational socialization goals (Vienna: *M *=2.8, *SD *=0.5; Kyoto: *M *=2.8, *SD *=0.5), *t*(65)=0.19, *p *=.424, *d *=0.05, post hoc t-tests, following a significant Socialization Goal * Culture interaction: *F*(1, 65)=5.30, *p *=.024, *pη² *=.08. However, mothers from both cultures emphasized autonomous over relational socialization goals, main effect Socialization Goal: *F*(1, 65)=13.72, *p* <.001, *pη² *=.64, main effect Culture: *F*(1, 65)=2.75, *p *=.102, *pη² *=.04. Note that this scale was originally developed without including a collectivistic dimension (which may have some but not all aspects in common with relational cultural contexts). Thus, we did not interpret or analyze data from these scales beyond this descriptive level.

Regarding attrition rates, 22 additional dyads (Kyoto: *n *=8; Vienna: *n *=14) were excluded from the analysis because infants did not complete the assessment (Kyoto: *n *=4; Vienna: *n *=3), moved excessively (Kyoto: *n *=2; Vienna: *n *=6), a technical error occurred with the video recording (Kyoto: *n *=2; Vienna: *n *=1), or because they did not provide sufficient trials for the analysis (Vienna: *n *=4, see EEG procedure for details). The targeted sample size was based on a previous study using a similar design (Köster et al., 2017). However, to account for the younger age and, consequently, higher attrition rates, we collected a much larger initial sample size (*n *=93).

### 2.2 Procedure and stimuli

#### 2.2.1 Warm-up phase

Families visited the EEG laboratory with their infants for one experimental session. In a brief warm-up phase, mothers in both cultural groups were asked to point out 3-5 different elements of a visual scene to their infants in a picture book (book title: “I Spy: A Book of Picture Riddles”; publisher: Scholastic), combined with verbal prompts (e.g., “Look at this!”) in their own mother tongue (German/Japanese). This served to train the mothers’ pedagogical pointing and check that infants competently followed their mothers’ pointing, which all infants did as confirmed by video annotation.

#### 2.2.2 EEG procedure

**Fig. 1.**
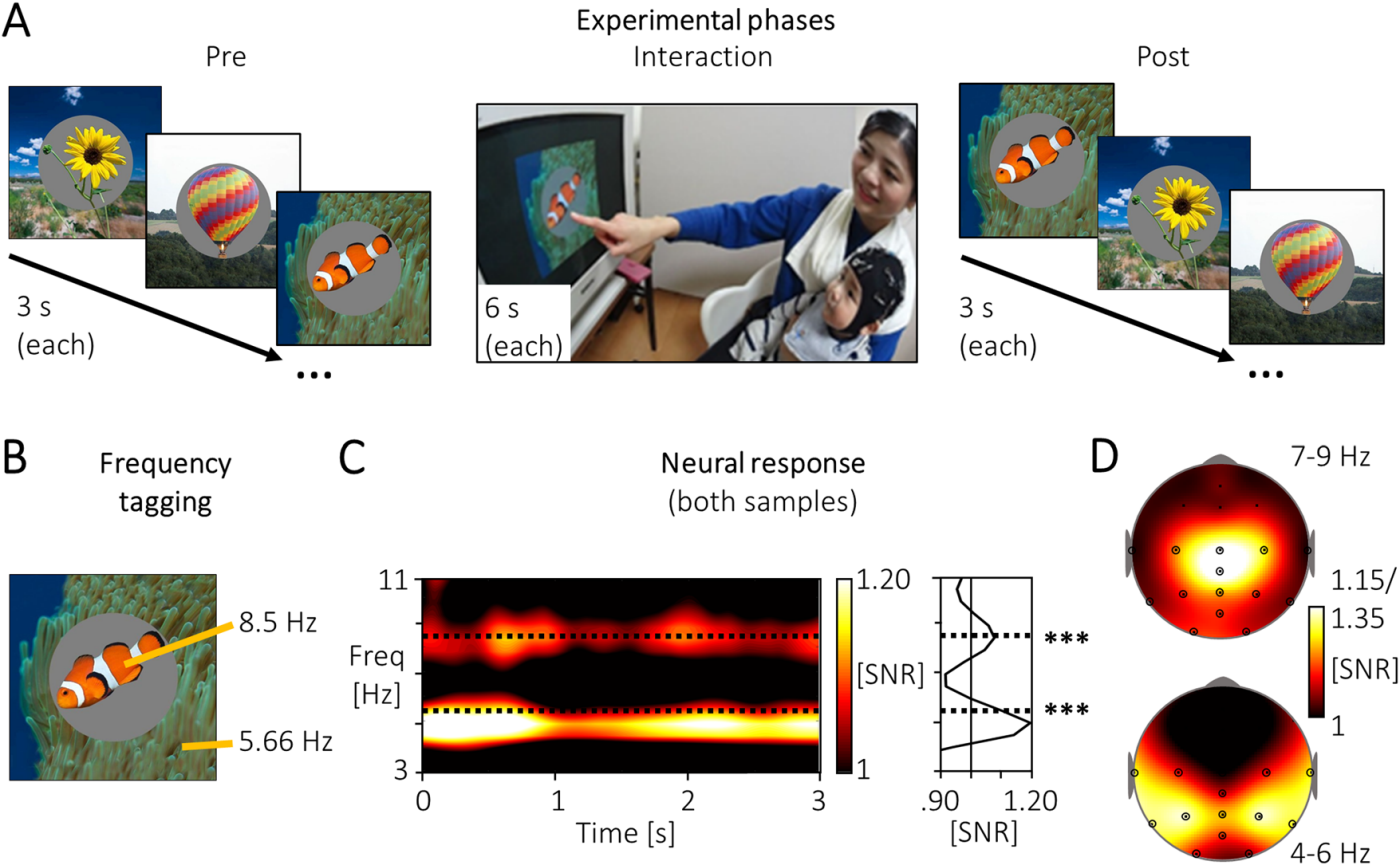
Experimental paradigm and grand mean neural response. (A) Twenty natural pictures with a clear object in front of a background were shown in a pre- and post-phase (3 s each) and during an interaction phase (6 s, each picture presented twice). In the interaction phase, the mother freely pointed out elements of the pictures to the infant. Before each image, a black screen was shown for EEG baseline recording (1 s) followed by an attention-getter, a white fixation dot with variable duration (0.5-1 s) accompanied by an infant-friendly sound. (B) By using frequency tagging, object and background were presented at different stimulation frequencies (5.67 or 8.5 Hz, counterbalanced), to elicit distinct neural signatures. (C) Frequency tagging led to a significant increase in the signal-to-noise ratio (SNR) compared to 1 at both stimulation frequencies (0-3000 ms, ***p <.001). (D) The SNR at the participants’ peak frequencies (ranges: 4-6 and 7-9 Hz; displayed up to 1.15 and 1.35 respectively) revealed the strongest signal at central, parietal, temporal, and occipital electrodes (black circles).

In the EEG paradigm, infants saw natural pictures of everyday animals and objects in their own environment, shown as the background (e.g., a fish in the sea or a bus on the road; retrieved from pixabay.com), while infants’ neural activity was recorded with a mobile EEG device. The stimuli selection was discussed between the authors from both cultural contexts and stimuli were chosen to be similarly familiar between cultures. The EEG session included an interactive part, where mothers pointed out elements of their choice in these pictures to their children, to test the maternal guidance of infants’ attention (see Fig. 1A). At the end of the session, mothers were asked to fill out a sociodemographic questionnaire and Keller’s socialization goals scale (Keller, 2007).

To ensure that the procedure was equivalent between cultures, all experimental procedures followed the same protocol, and an identical setup with the same EEG system (SMARTING, mBrainTrain, Serbia) and video camera (SONY ActionCam, Sony, Japan) were used in both cultures, except the computers and CRT monitors used for stimulus presentation and neural data recording. The portable EEG system and video camera were first brought to Japan, and then to Austria. The similarity of testing conditions was further ensured by a visit of the experimenter (AB) who conducted the data assessment in Vienna at the laboratory in Kyoto, where she accompanied the data assessment of *n *=22 dyads.

During the EEG procedure, infants sat on their mothers’ lap at a distance of 70-90 cm from the screen. We applied a pre-post design. In the pre- and post-phases, the infant watched the pictures individually, with the mother-infant interaction phase in between. In the pre-interaction phase (pre-phase), infants saw 20 different natural pictures with a focal object in front of a background (for all pictures, see Fig. S1), shown for 3 s. Mothers were instructed to quietly watch the pictures together with their infants. In the interaction phase, the same 20 images were presented twice (i.e., each picture was repeated immediately, in the consecutive trial), for a total of 6 s per trial. Mothers were asked to point out elements of the pictures to their infants, that they considered interesting, preferably a different element for each picture when shown the second time. Mothers pointed with their index finger, combined with a short verbal comment (e.g., “Look at this!”), as trained in the warm-up phase, and were asked to keep the finger on the screen until the stimulus disappeared from the screen. Mothers were further instructed to avoid naming the animals and objects shown on the screen. In the post-interaction phase (post-phase), the same 20 images were presented again, for 3 s each, while the mother and infant watched the screen quietly. This design resulted in 80 trials; each image being presented a total of four times to each dyad across all phases (20 different images in the pre-phase, 2 × 20 in the interaction phase, 20 in the post-phase).

#### 2.2.3 Stimuli

We applied a frequency tagging method (Müller et al., 2003) to track infants’ neural responses to object and background, when presented simultaneously (Fig. 1B). Specifically, to elicit separate neural signatures for object and background, the object was presented at 5.67 Hz and the background was presented at 8.5 Hz, or vice versa. This was achieved by controlling the presentation of an 85 Hz CRT monitor at every refresh cycle (Köster et al., 2017), implemented in Psychophysics Toolbox (version 3.0; MATLAB R2018b). For example, for a flicker rate of 8.5 Hz for the object, the object was presented at a duty cycle of 5:5, that is, five refresh cycles with the object being illuminated (100% brightness) and five refresh cycles with the object being darkened (20% of the original brightness). A non-flickering gray circle was included between the object and the background to avoid a shadow of the object, which would be present in the background element of the stimulus and may interfere with the frequency tagged for the background. To further avoid the gray circle being perceived as a separate element of the stimulus, we included a white fixation spot at the exact size of the gray circle, which then changed into the gray circle upon stimulus onset. This way, perceptually, the white fixation spot seemed to disappear rather than appear as an additional element of the stimulus.

Each image was presented following a black screen (1 s) and a white fixation dot with the size of the gray circle separating the object and background (variable duration of 0.5-1 s), accompanied by an infant-friendly sound (five distinct sounds were used in a randomized order). The pictures were presented at a visual angle of about 12.5 × 12.5. This visual angle is covered by the central visual field, such that the whole picture was visually processed, independent of the gaze position on the picture. The image order was randomized for each participant and for each of the phases. Frequency combinations were counterbalanced, such that participants saw both frequency combinations (5.67 Hz for object, 8.5 Hz for background, and vice versa) in all three phases (pre, interaction, post) but kept identical for the specific stimulus pictures across the phases. Such counterbalancing controls for the power differences elicited by the two different driving frequencies (see EEG analysis). (Note: In Kyoto, we additionally counterbalanced the frequency combination [i.e., object presented at 5.67 or 8.5 Hz] of the specific stimuli between participants. However, as confirmed by control analyses, this minor difference in the stimulation procedure did not have an effect on any of the results.)

In case infants’ attention decreased during the stimulus presentation, an infant-friendly animation (a black spiral turning in front of a white background, accompanied with music) was inset between trials by the experimenter who monitored infants’ attention via a remote live-view wireless display device of the video camera. If the infant became fussy, the presentation was paused or stopped. Dyads were video recorded during the EEG assessment for the subsequent coding of infants’ gaze behavior and maternal pointing.

#### 2.2.4 EEG apparatus and analysis

At both recording sites, infants’ brain activity was recorded with the same mobile EEG system (SMARTING, mBrainTrain, Serbia) from 24 Ag/AgCl scalp electrodes (EasyCap GmbH, Germany), at a sampling rate of 500 Hz. Impedances were kept as low as possible, aiming for values below 10 kΩ.

All EEG data analyses and visualization were conducted in MATLAB (MathWorks Inc., US, Version R2018b) using the EEGLAB toolbox (Delorme & Makeig, 2004, Version 2019) and custom-made scripts. Prior to the analysis, continuous EEG data were band-pass filtered from 1 to 70 Hz and then segmented into epochs from -1000 to 4000 ms (pre- and post-phase) or -1000 to 7000 ms (interaction phase) with regard to the stimulus (picture) onset. For the pre- and post-phase, we removed all trials, in which the infant did not watch at least 2500 ms of the trial (3000 ms), looking towards the screen at least 500 ms after stimulus onset, or in which the infant looked away before stimulus offset (coded from video). This decision was made to retain as many trials as possible and because the power of the object and background signal is controlled within each trial (i.e., those times that the infant looks away none of the signals is present). Noisy trials were removed, and bad channels were visually identified and removed based on visual inspection. Eye blinks and muscle artifacts were then detected using an independent component analysis procedure and removed after visual inspection. Optionally, some additional trials and channels were removed (up to 3 scalp channels in total). Finally, electrodes were re-referenced to the average of all scalp electrodes. For the interaction phase, we only selected those trials, in which the mother pointed within the first 3000 ms, and the infant watched at least 2000 ms of the last 3000 ms of the trials and applied the same preprocessing steps.

In the analysis of the pre- and the post-phase, we included those infants with at least one clean trial for both frequency combinations (object at 5.67 Hz, background at 8.5 Hz, and vice versa) of both phases (pre and post). Infants included in the analysis provided on average *M *=21.7 (*SD *=5.8; *range *=11-33) and *M *=27.9 (*SD *=6.7; *range *=12-40) trials, for Vienna and Kyoto, respectively.

For the analysis of the interaction phase, we aimed to include those infants with at least one clean trial for both frequency combinations (object at 5.67 Hz, background at 8.5 Hz, and vice versa) in both maternal pointing conditions (object, background). However, the distribution of object and background points was highly unequal (i.e., 20 mothers across the two samples only pointed to the object), and infants were quite active, leading to an overall low quality of the EEG data from the interaction phase. Because only 14 infants from Vienna and 24 infants from Kyoto would have remained in this analysis, we did not further analyze the EEG data from the interaction phase.

To obtain the evoked spectral power over time, the trial data were averaged across the trials of the two distinct frequency combinations (object at 5.67 Hz, background at 8.5 Hz, and vice versa) for each phase (pre, post), separately. For example, we averaged all trials of the pre-phase, with the object presented at 5.67 Hz and the background presented at 8.5 Hz. These event-related potentials were then analyzed using Morlet wavelets (Tallon-Baudry & Bertrand, 1999) with approximately 7 cycles, in 0.5 Hz steps in the frequency range of 1-15 Hz. Afterwards, we combined the mean activity at the object and background frequency, across both frequency conditions. For example, the object amplitude for the pre-phase is composed of the power for objects when presented at 5.67 and 8.5 Hz, with an equal weight. Note that this approach effectively controls for the potential power differences between the lower and the higher frequencies.

In a first step, we optimized the detection of the two stimulation frequencies, by calculating the time-resolved signal-to-noise ratio (SNR): For each frequency band and time point (i.e., the result from the wavelet analysis), we divided the spectral power at this time point by the average power of the surrounding frequencies (±1, ±2 Hz, around the target frequency), across the whole time window (-1000 to 4000 ms), as a proxy for the noise level. The resulting SNR (see Fig. 1C) revealed a peak between 4-6 Hz for the lower driving frequency (5.67 Hz) and a peak between 7-9 Hz for the higher driving frequency (8.5 Hz). Note that we applied a wavelet analysis approach similar to our previous study, establishing the method (Köster et al., 2017). We used SNR values rather than a pre-stimulus baseline, because of the lower trial numbers and lower frequencies used in the present study, leading to more variability in the baseline activity, than in our previous EEG study with older children. To statistically test whether we successfully stimulated infants’ brain activity, we used one-sample t-tests, comparing the grand mean SNR values (across both cultures and all conditions, 0-3000 ms, electrodes marked in Fig. 1D) against the noise level of 1, at the wavelets which were closest to the stimulation frequencies, namely 5.5 and 8.5 Hz, revealing a signal significantly higher than noise levels (see Fig. 1C).

Because the peak frequencies varied clearly between individuals, within the 4-6 and 7-9 Hz range (which was somewhat below the actual driving frequencies), we determined individual frequencies as the maximal SNR value in these frequency ranges across all central, parietal, temporal, and occipital channels (Cz, C3, C4, CPz, Pz, P3, P4, P7, P8, T7, T8, POz, O1, O2; see Fig. 1D), and the whole trial duration (0-3000 ms). (The individual frequencies between 4-6 Hz were, Vienna: *M *=4.58, Kyoto: *M *=4.94, and between 7-9 Hz, Vienna: *M *=8.16, Kyoto: *M *=8.14). These individual SNR values were then used for all subsequent analysis steps.

**Fig. 2.**
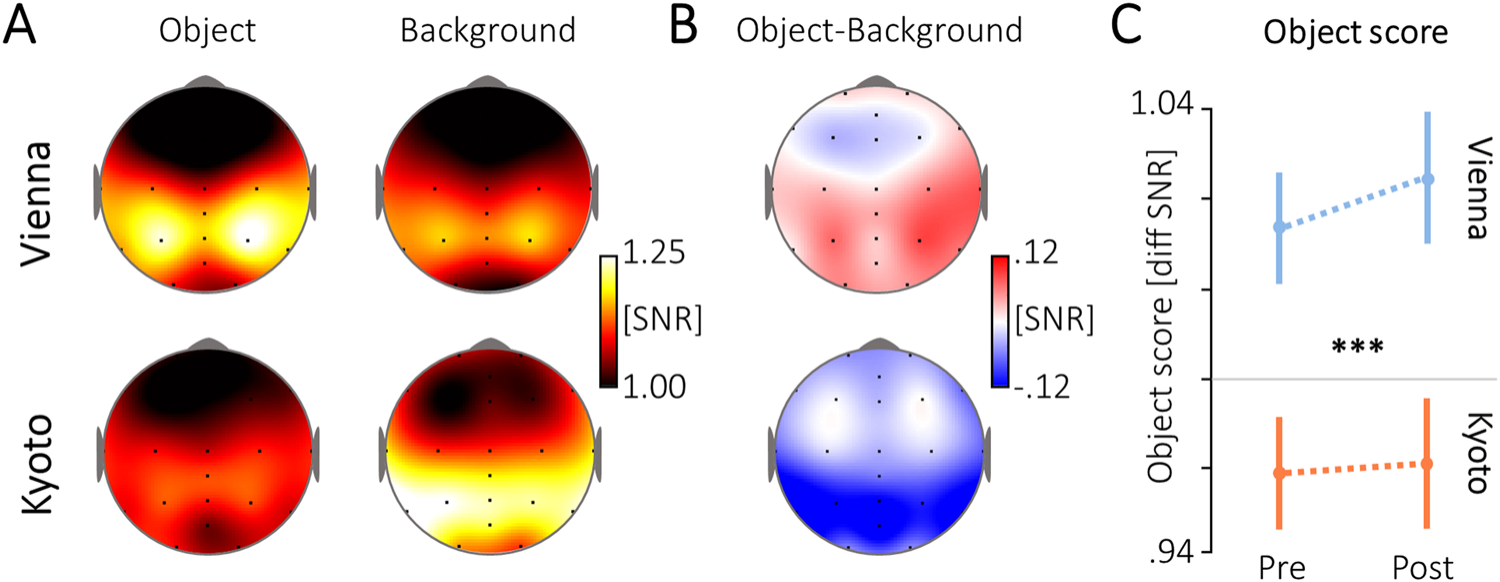
Topographies for (A) the object, the background, and (B) the object-background difference in the neural response of infants from Vienna and Kyoto. Values indicate the signal-to-noise ratio (SNR) at individualized frequencies, averaged across both phases (pre, post), for the whole time window of stimulus presentation (0-3000 ms). (C) The relative activity for the object versus the background (object score, for the electrodes marked in Fig. 1D), calculated as the SNR of the object, divided by the mean SNR of the object and background (whiskers indicate standard errors, the gray line at 1.00 corresponds to an equal signal strength for object and background), main effect Culture: ***p <.001.

For the topographies of the object and background signal for both cultural groups (Vienna, Kyoto; as shown in Fig. 2A), we calculated the grand mean signal for the object or background (individual SNR values), in the pre- and post-phase, at the specific object or background frequency (e.g., the object activity would be the mean of a combination of the pre/4-6 Hz, post/4-6 Hz, pre/7-9 Hz, and post/7-9 Hz trials, in which the object was presented at the specific frequency). For the object-background difference topography, the difference between the object and background signal was taken. For the statistical comparison of the activity between cultural groups, as well as between the pre- and the post-phase, an object score was calculated for the pre- and post-phase separately, as the object activity divided by the mean of the object and background activity (reflecting the relative SNR between object and background). The relative SNR values were entered into a mixed model ANOVA, with Culture (Vienna, Kyoto) as a between-subject factor and Phase (pre, post) as a within-subject factor. Statistics were calculated in SPSS (version 28.0.0.0, IBM).

#### 2.2.5 Maternal pointing analysis

Maternal pointing behavior during the interaction phase was video recorded and the pointing to object or background was analyzed using Interact (Mangold International GmbH., Germany, 2018). We coded maternal pointing toward the object, the background, and invalid points (i.e., pointing was unclear, or the mother missed to point). We established the interrater reliability between two independent coders for >20% of the data (10 dyads in each context, drawn from the initial sample; Kyoto: κ =0.96, Vienna: κ = 0.97). For the statistical analysis of maternal pointing, we calculated the percentage of valid points towards the object (number of object points/number of all valid points) and carried out a t-test between samples in RStudio (packages ggplot2, ggpubr).

## 3 Results

### 3.1 SNR at the stimulation frequencies

The grand mean activity, across the pre- and post-phase, revealed clear SNR (>1) at the stimulation frequencies 5.67 and 8.5 Hz (Fig. 1C), *t*(70) = 4.73, *p* <.001, *d* = 0.56, and, *t*(70) = 8.17, *p* <.001, *d* = 0.96. This activity was most prominent at central, parietal, temporal, and occipital electrodes (marked in Fig. 1D), with a somewhat more centralized response to the higher compared to the lower frequency (Fig. 1D; see topographies). This neural response pattern and topography was consistent between both cultures, Vienna and Kyoto (see Fig. S2). Based on these grand mean topographies, the electrodes marked in Figure 1D were used in all further analyses.

### 3.2 Infants’ object and background processing

Infants from Vienna showed a higher SNR for object versus background elements, while infants from Kyoto showed a higher SNR for background versus object elements (Fig. 2A), across the pre- and post-phases (Fig. 2B): Culture * Phase ANOVA on the neural object score (i.e., the relative SNR for the object versus the background) across the whole time window (0-3000 ms), main effect Culture: *F*(1, 69) = 18.85, *p* <.001, *pη²* = .22. However, we did not find any difference in infants’ neural object score between the pre- and the post-phase, main effect Phase: *F*(1, 69) = .24, *p* = .622, *pη²* <.01, nor any Culture * Phase interaction: *F*(1, 69) = .11, *p* = .735, *pη²* <.01.

This analysis was conducted with optimized SNR values at individual frequencies (based on the grand mean activity, independent of conditions; see details on EEG analysis in the online methods). To test whether the main results would rely on the SNR measure, we conducted a complementary analysis, analyzing the raw amplitude values at the wavelets closest to the stimulation frequencies (i.e., the wavelets at 5.5 and 8.5 Hz; without taking any baseline) in the same way as the individualized SNR values. This analysis confirmed our main results (see Fig. S3), namely a higher object score in Vienna compared to Kyoto, main effect Culture: *F*(1, 69) = 24.25, *p* <.001, *pη²* = .26, but no difference in infants’ neural object scores between the pre- and the post-phase, or any interaction between Culture and Phase, main effect Phase: *F*(1, 69) = 1.89, *p* = .174, *pη²* = .03, Culture * Phase interaction: *F*(1, 69) = .07, *p* = .792, *pη²* <.01.

### 3.3 Maternal pointing

**Fig. 3.**
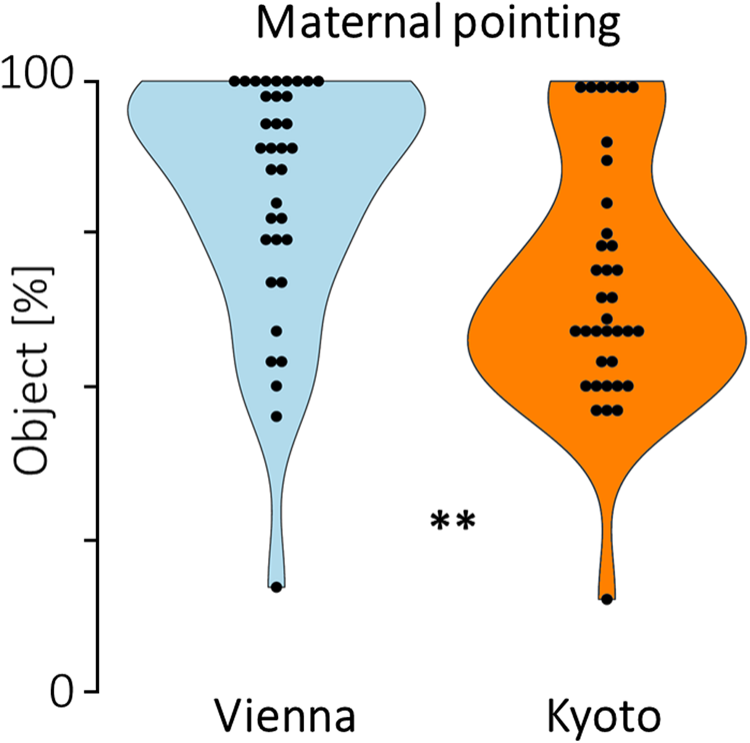
Maternal pointing to the object versus the background. Violin plots indicate the proportion of points that mothers made to the object, and dots indicate individual participants. Mothers from Vienna pointed more often to the object than mothers from Kyoto, **p = .002.

We analyzed the proportion of trials in which mothers pointed to the object versus background elements of the presented visual scenes in the interaction phase. We found a clear difference between cultures (Fig. 3): Mothers from Vienna pointed more frequently to the objects (*M* = 82%, *SD* = 20.0) than mothers from Kyoto (*M* = 66.7%, *SD* = 19.9), *t*(69) = 3.27, *p* = .002, *d* = .78.

We then tested the relation between maternal pointing behavior in the interaction phase (object score in %) and changes in the object score from pre- to post-phase (the difference in the object score between the pre- and post-phase), by including maternal pointing behavior and cultural group as two predictors in a regression model. Both predictors were non-significant, maternal pointing: *β* = .024, *p* = .725, culture: *β* = -.005, *p* = .854. At the level of individual samples, there was a marginal correlation between maternal points toward the object and infants’ increase in object scores from pre- to post-phase in the sample from Vienna, *r* = .33, *p* = .053, but no such effect was found in the sample from Kyoto, *r* = -.19, *p* = .266.

## 4 Discussion

We report cultural differences in the processing of objects versus background elements in visual cortical networks of 12-month-olds. This is several years earlier than previously thought (Imada et al., 2013; Köster et al., 2017), based on verbal and/or behavioral measures of context-sensitivity, and underscores a substantial role of culture in the early development of the human visual system. These findings complement former studies on environmental impacts on early visual processing (Banks et al., 1975; Berardi et al., 2000; Emberson et al., 2015) and cross-cultural differences in visual exploration patterns in social contexts (Astor et al., 2022; Bard et al., 2021; Geangu et al., 2016; Green et al., 2016; Köster et al., 2020; Haensel et al., 2020; Kärtner et al., 2010; Keller, 2007), making the case for cross-cultural differences in the visual processing of the physical environment in the infant years.

It is a central assumption that social interactions with close others form a key mechanism of early cultural transmission and learning (Csibra & Gergely, 2009; Shneidman & Woodward, 2016; see also Bánki et al., 2023, for a recent RVS study). Here, we provide further evidence for cross-cultural differences in parent-infant interactions. Mothers from Vienna pointed out object (versus background) elements to their infants more frequently than mothers from Kyoto during social interaction. The cross-cultural difference in parental pointing is in line with former research with parents and older children (Senzaki & Shimizu, 2020; Senzaki et al., 2016), as well as initial evidence that parental guidance of children’s attention scaffolds the development of human visual cognition (Köster & Kärtner, 2018; Senzaki et al., 2016), and brain development more generally (Huber et al., 2023).

However, we only found weak evidence for the direct effects of parental guidance of attention on the changes in object versus background processing from the pre- to the post-phase in the subsample from Vienna, but no such effect was present in the subsample from Kyoto. That we did not find a clear effect of parental guidance on infants’ visual processing may be due to several aspects of the present study design. Given the constraints of EEG experiments with infants, the interaction phase between pre- and post-test was very brief and relatively fuzzy due to participants’ movement. Furthermore, although maternal behavior was different between cultures, the proportion of maternal pointing to the object was still in a similar range (i.e., between 50-100% towards the object), which further limits the potential impact of these short social interactions in an in-lab and non-naturalistic setting. Additionally, the similarity of the EEG findings from the pre- and the post-phase points to a more persistent and already existing effect of culture on visual attention development. Thus, the present study does not yield conclusive evidence regarding cross-cultural differences in the influence of parental attention guidance on infants’ visual processing. This leaves room for further investigations (e.g., longitudinal, or experimental studies) but also additional alternative explanations (e.g., genetic factors or gene-culture co-evolution accounts). Existing findings also highlight the influence of culture-specific visual environments on infants’ visual attention (e.g., Kuwabara et al., 2020), while studies with older children found links between visual attention to objects and language input (Waxman et al., 2016), or context-sensitivity and communication style (Schulze et al., 2022).

Although it has been shown previously that visual cortical processing is profoundly shaped by experience in humans (e.g., Berardi et al., 2000; Emberson et al., 2015) and other mammalian species (e.g., Banks et al., 1975), this is the first study to show cross-cultural differences in early visual cortical development. This has been possible by combining a cross-cultural neuroscience approach and frequency tagging (Martens et al., 2011; Müller et al., 2003). A cross-cultural approach to studying early brain development holds the potential to reveal how complex structures of the environment influence early human neural and cognitive development. The frequency tagging approach applied here is particularly fruitful in studying early visual cortical development because it captures visual processes more directly than conventional behavioral approaches such as eye-tracking (Köster et al., 2023; Norcia et al., 2015; Vialatte et al., 2010). It further allows us to capture visual processes more directly, in terms of overt and covert attention and the processing in visual cortical networks to several stimuli presented simultaneously (Christodoulou et al., 2018; Robertson et al., 2012). Future investigations may further benefit from the decoding of perceptual elements from the infant brain (Xie et al., 2022) or by linking structural brain development to early experiences across diverse cultures.

To conclude, this study grounds the ontogenetic origins of cultural impacts on human visual processing in the first year after birth, for the showcase of object and background processing. This is much earlier than documented to date, using conventional (verbal or behavioral) measures of object and background perception. This study demonstrates the unique potential of a cross-cultural developmental neuroscience approach in uncovering the early foundations of human cognitive development. Our results emphasize that we are just at the beginning of understanding human early neuro-cognitive development, situated in the complex and culturally diverse visual, haptic, and social environments young infants grow up in.

## Supplementary Material

Supplementary Material

## Data Availability

All stimulus materials and raw data are available in a permanent online repository (https://doi.org/10.17605/OSF.IO/R82NM). Furthermore, analysis scripts will be made available upon request.
