# Supplementary material for: Cross-cultural differences in visual object and background processing in the infant brain
Source: Imaging Neurosci (Camb). 2023 Dec 13;1:imag-1-00038. doi: 10.1162/imag_a_00038 (PMC12007513; doi:10.1162/imag_a_00038)
Supplement: Supplementary Material [file imag_a_00038-supp.pdf]

Cross-cultural differences in visual object and background processing in the infant brain

Moritz Köster, Anna Bánki, Daiki Yamasaki, Masaharu Kato, Shoji Itakura, & Stefanie Hoehl

Imaging Neuroscience, 2023

Supplementary Material

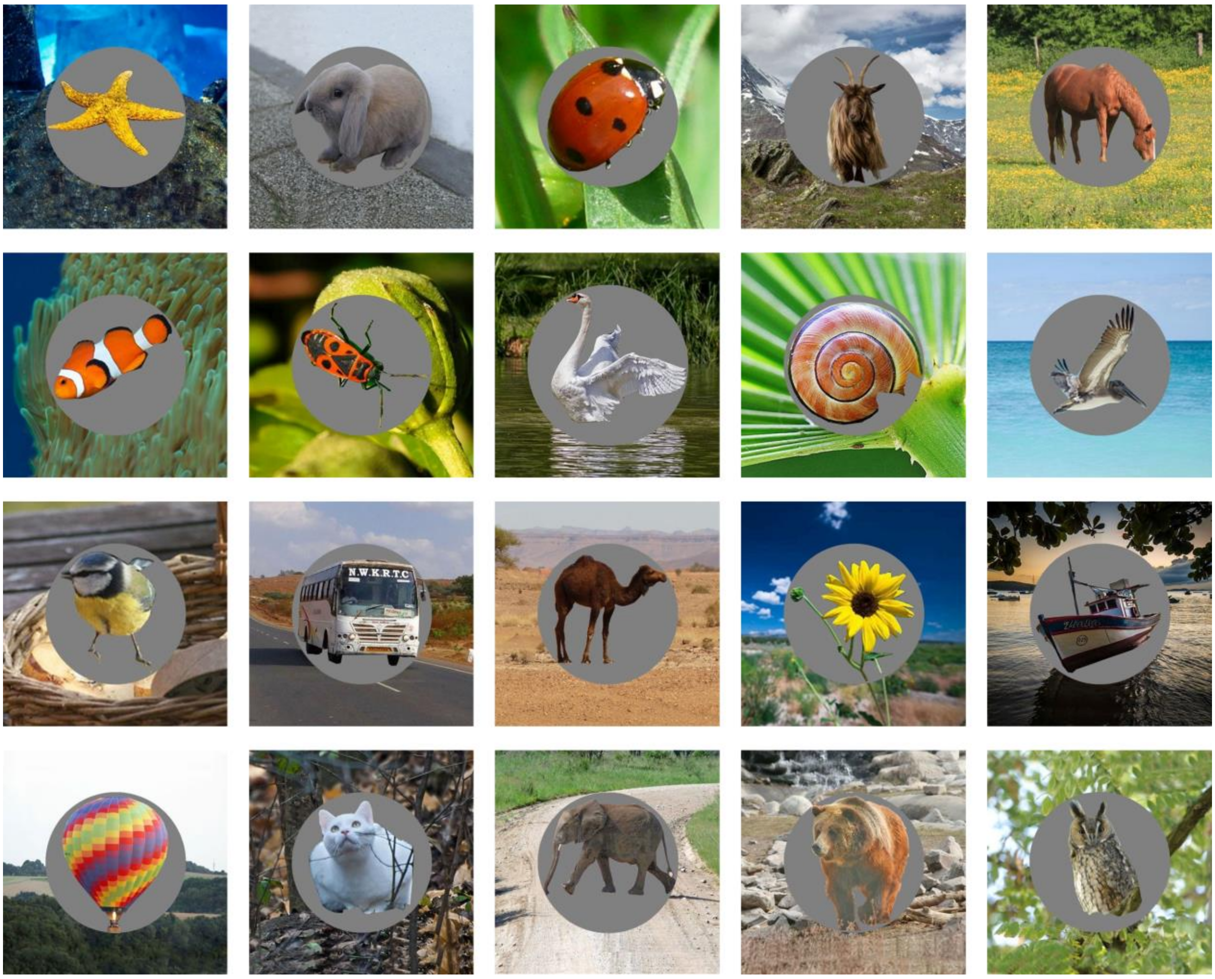

Figure S1. Overview of all stimuli images (visual scenes with natural objects in front of a natural background).

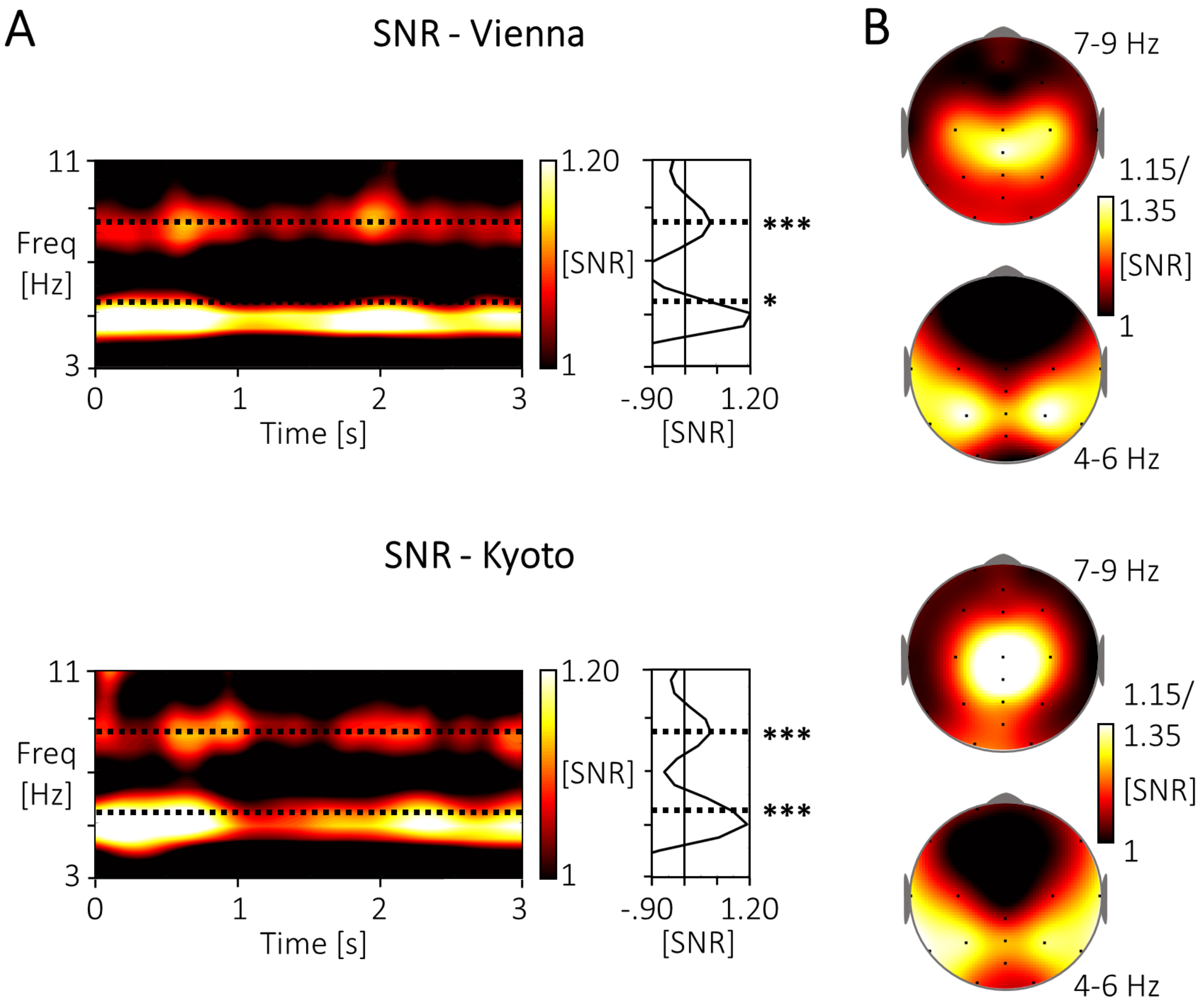

Figure S2. Neural response to frequency tagging, split by cultural groups. (A) In both cultural groups, the two stimulation frequencies led to an increase in the signal-to-noise ratio (SNR) at the stimulation frequencies of 5.67 and 8.5 Hz, across the whole time window (0-3000 ms, * p = .037, *** $p \leq$ .001). (B) The SNR at the participants' individual peak frequencies in the 4-6 and 7-9 Hz ranges (across stimulation conditions), split by cultural groups (the grey line at 1.00 corresponds to equal signal strength for object and background).

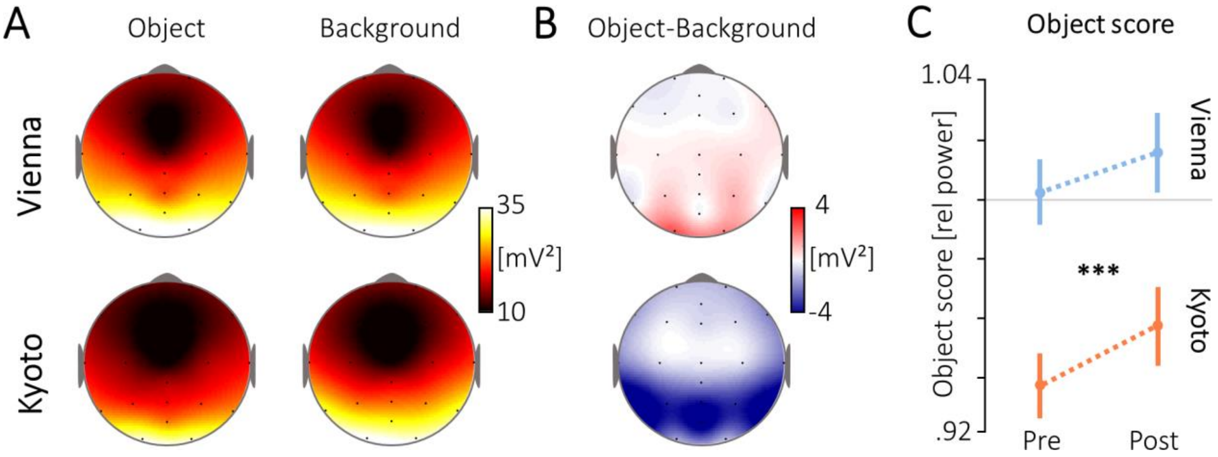

Figure S3. Main analysis with the raw signal power, instead of the individual SNR values. (A) Topographies for the object, background, and (B) object-background difference in neural activity for infants from Vienna and Kyoto, across both phases (pre and post). Values indicate the raw signal power at the specific stimulation frequencies of 5.5 and 8.5 Hz, for the whole time window of stimulus presentation (0-3000 ms). (C) The relative activity for the object versus the background (object score) was higher in Vienna, compared to Kyoto, independent of the experimental phase (pre, post), main effect Culture: *** p = .001.
